# Possible Role of GADD45γ Methylation in Diffuse Large B-Cell Lymphoma: Does It Affect the Progression and Tissue Involvement?

**DOI:** 10.4274/tjh.2014.0174

**Published:** 2015-12-03

**Authors:** İkbal Cansu Barış, Vildan Caner, Nilay Şen Türk, İsmail Sarı, Sibel Hacıoğlu, Mehmet Hilmi Doğu, Ozan Çetin, Emre Tepeli, Özge Can, Gülseren Bağcı, Ali Keskin

**Affiliations:** 1 Pamukkale University Faculty of Medicine, Department of Medical Biology, Denizli, Turkey; 2 Pamukkale University Faculty of Medicine, Department of Medical Pathology, Denizli, Turkey; 3 Pamukkale University Faculty of Medicine, Department of Hematology, Denizli, Turkey; 4 Pamukkale University Faculty of Medicine, Department of Medical Genetics, Denizli, Turkey

**Keywords:** GADD45γ, DNA methylation, Diffuse large B-cell lymphoma

## Abstract

**Objective:**

Diffuse large B-cell lymphoma (DLBCL) is the most common type of non-Hodgkin lymphoma among adults and is characterized by heterogeneous clinical, immunophenotypic, and genetic features. Different mechanisms deregulating cell cycle and apoptosis play a role in the pathogenesis of DLBCL. Growth arrest DNA damage-inducible 45 (GADD45γ) is an important gene family involved in these mechanisms. The aims of this study are to determine the frequency of GADD45γ methylation, to evaluate the correlation between GADD45γ methylation and protein expression, and to investigate the relation between methylation status and clinicopathologic parameters in DLBCL tissues and reactive lymphoid node tissues from patients with reactive lymphoid hyperplasia.

**Materials and Methods:**

Thirty-six tissue samples of DLBCL and 40 nonmalignant reactive lymphoid node tissues were analyzed in this study. Methylation-sensitive high-resolution melting analysis was used for the determination of GADD45γ methylation status. The GADD45γ protein expression was determined by immunohistochemistry.

**Results:**

GADD45γ methylation was frequent (50.0%) in DLBCL. It was also significantly higher in advanced-stage tumors compared with early-stage (p=0.041). In contrast, unmethylated GADD45γ was associated with nodal involvement as the primary anatomical site (p=0.040).

**Conclusion:**

The results of this study show that, in contrast to solid tumors, the frequency of GADD45γ methylation is higher and this epigenetic alteration of GADD45γ may be associated with progression in DLBCL. In addition, nodal involvement is more likely to be present in patients with unmethylated GADD45γ.

## Abstract (tr)

**Amaç:**

Diffüz büyük B-hücreli lenfoma (DBBHL) yetişkin bireylerde Hodgkin-dışı lenfomaların en yaygın tipidir ve klinik, immünofenotipik ve genetik özellikler açısından heterojen özellikler taşıması ile karakterizedir. DBBHL patogenezinde hücre döngüsü ve apoptoz regülasyonunu bozan farklı mekanizmalar rol oynamaktadır. Growth arrest DNA damage-inducible 45 (GADD45γ), bu mekanizmalarda yer alan önemli bir gen ailesidir. Bu çalışmanın amaçları DBBHL doku örnekleri ve reaktif lenfoid hiperplazili bireylerin reaktif lenfoid doku örneklerinde GADD45γ metilasyon sıklığını belirlemek, GADD45γ metilasyonu ile protein ekspresyonu arasındaki ilişkiyi değerlendirmek ve DBBHL olgularında metilasyon durumunun klinikopatolojik parametrelerle ilişkisini araştırmaktır.

**Gereç ve Yöntemler:**

Bu çalışmada 36 adet DBBHL doku örnekleri ve 40 adet malign-olmayan reaktif lenfoid doku örnekleri analiz edildi. GADD45γ metilasyon durumunu belirlemek için metilasyona-duyarlı yüksek çözünürlüklü erime eğrisi analizi kullanıldı. GADD45γ protein ekspresyonu immünohistokimyasal analiz ile belirlendi.

**Bulgular:**

DBBHL’de GADD45γ metilasyonunun sık olduğu belirlendi (%50). Aynı zamanda, erken evre ile karşılaştırıldığında ileri evre tümörlerde GADD45γ metilasyonu istatistiksel olarak anlamlı düzeyde yüksekti (p=0,041). Ancak, GADD45γ metilasyon yokluğunun primer anatomik yerleşim olarak nodal tutulumla ilişkili olduğu belirlendi (p=0,040).

**Sonuç:**

Bu çalışmanın sonuçları solid tümörlerin aksine, DBBHL’de GADD45γ metilasyon sıklığının yüksek olduğunu ve GADD45γ geninde gözlenen bu epigenetik değişimin, hastalığın progresyonu ile ilişkili olabileceğini göstermektedir. Buna ek olarak, nodal tutulum daha çok GADD45γ metile olmayan olgularda gözlenmektedir.

## INTRODUCTION

Diffuse large B-cell lymphoma (DLBCL) is the most common group of non-Hodgkin lymphomas (NHLs) and represents 30% to 40% of all newly diagnosed NHLs in Western countries. DLBCL represents a heterogeneous group of neoplasms with diversity in clinical presentation, morphology, and genetic and molecular properties [1]. It is well known that genetic and epigenetic changes that create a difference in gene expression profiles between normal and malign B cells are responsible for the heterogeneity of DLBCL. Genetic aberrations in DLBCL are chromosomal translocations, aberrant somatic hypermutations, and copy number variations including amplifications or deletions [2,3,4,5]. Other differences come from epigenetic modifications such as DNA methylation [6,7,8].

DNA methylation may lead to transcriptional silencing by at least 3 different mechanisms: inhibition of binding of the transcription factors to their specific sequences, a direct effect on nucleosome positioning, and recruitment of other nuclear factors that recognize the methylated CpG dinucleotide blocks binding other factors including transcription factors [9]. To date, a number of genes involved in the regulation of DNA repair, cell cycle control, and apoptosis, such as MGMT [10,11], DAPK1 [12], and GADD45γ [13], have been determined as hypermethylated in DLBCL. A recent study also showed that abnormal methylation patterns might be seen depending on chromosomal regions, gene density, and methylation status of neighboring genes in normal B-cell populations and NHL [8].

The growth arrest DNA damage-inducible (GADD45) gene family plays important roles in various cell functions such as DNA repair, cell-cycle control, and cell growth [14]. The members of the GADD45 gene family, GADD45γ, GADD45γ, and GADD45γ, are evolutionarily conserved and expressed in both fetal and adult tissues [15,16,17]. They act as stress sensors that modulate cellular response against various physical and environmental stress factors [14,17,18]. It is also suggested that GADD45 proteins may provide a link between DNA repair mechanisms and chromatin remodeling [19,20]. Although all 3 proteins have similar functions, these functions are not identical since they have different activation pathways depending on cell type and the source of the stress [17,21].

There are very limited data in the literature about the role of GADD45γ in DLBCL pathogenesis. In this study, we aimed to show the methylation status and expression profiles of GADD45γ in DLBCL tissues and nonmalignant reactive lymphoid node tissues (RLTs). We also focused on the relationship between GADD45γ methylation status and clinicopathologic parameters of DLBCL.

## MATERIALS AND METHODS

### Tissue Samples

We analyzed 36 DLBCL tissue samples and 40 nonmalignant RLTs that were diagnosed in the Department of Pathology of Pamukkale University between 2009 and 2012. Tissue samples were collected from all patients before treatment. Based on Hans’s algorithm, DLBCL cases were classified as germinal center (GC) and non-GC in the Pathology Department [22]. All of the patients with DLBCL were also classified by Ann Arbor stage and International prognostic index (IPI) score according to the previously described criteria [23,24]. This study was approved by the Institutional Review Board of Pamukkale University and was in compliance with the Declaration of Helsinki.

Two consecutive sections of formalin-fixed and paraffin-embedded (FFPE) tissues were used for DNA isolation and immunohistochemistry (IHC). DNA was isolated using a commercial kit according to the instructions of the manufacturer (QIAamp DNA Mini Kit, QIAGEN, the Netherlands) and IHC was performed using polyclonal antibody against GADD45γ as described previously [25].

### Methylation-Sensitive High-Resolution Melting Analysis

DNA samples underwent bisulfite treatment prior to methylation-sensitive high-resolution melting (MS-HRM) analysis by use of a commercial kit (EZ DNA Methylation-Gold Kit, Zymo Research, USA). Forward and reverse primers were as follows, respectively: 5’-CGTCGTGTTGAGTTTTGGT and 5’-TAACCGCGAACTTCTTCCA [26]. The protocol for identification of the amplicon by MS-HRM analysis is given in Table 1. For the confirmation of melting temperature (Tm) degrees in MS-HRM analysis, commercially available control DNA samples were used (EpiTect Control DNA Set, QIAGEN). All analyses were performed on a LightCycler 480 instrument (Roche Diagnostics, Germany).

### Immunohistochemistry

All immunostaining procedures including deparaffinization and antigen retrieval processes were performed automatically using the BenchMark XT automated stainer (Ventana Medical Systems, USA). GADD45γ (dilution: 1/200, Bioss Laboratories, USA) was used as the primary antibody. Larynx squamous cell carcinoma tissue samples were used as positive controls while negative controls were treated with the same IHC method by omitting the primary antibody. Granular cytoplasmic staining was assessed as positive. Immunohistochemical status of GADD45γ was scored as 0 (less than 25% positive cells), + (26% to 50% positive cells), ++ (51% to 75% positive cells), or +++ (more than 75% positive cells).

### Statistical Analysis

The methylation status and protein expression level of GADD45γ between DLBCL patients and RLT controls was compared using the chi-square test. The Fisher’ exact test was used to compare the protein expression and methylation of GADD45γ. The age-adjusted frequency ratios of GADD45γ methylation were calculated using multiple logistic regression analysis. P<0.05 was considered to be statistically significant.

## RESULTS

### Clinicopathologic Parameters

The median ages were 67.5 (range: 24-80) and 28.00 (range: 1-79) years in DLBCL patients and RLT controls, respectively. The most frequent sites of extranodal involvement were as follows when the patients were classified according to the anatomic site of tumor: lung (6 cases, 42.9%), bone marrow (3 cases, 21.4%), liver (2 cases, 14.3%), and stomach (2 cases, 14.3%).

### GADD45γ Methylation

**Figure 1:**
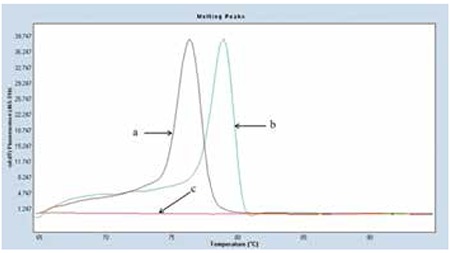
The high-resolution melting curves for GADD45γ methylation in diffuse large B-cell lymphoma, patients. a. Unmethylated GADD45γ DNA had a melting peak at 76±0.5 °C, b. Methylated GADD45γ DNA had a melting peak at 79±0.5 °C, c. Negative control (PCR-grade water was used instead of template DNA).

The Tm was 79±0.5 °C in the methylated region of the GADD45γ gene while the unmethylated region had a Tm of 76±0.5 °C in MS-HRM analysis, which was also confirmed by the control DNA samples. According to this finding, GADD45γ methylation was present in 18 of the DLBCL patients (50%), whereas 16 (40%) of the controls were methylated (Table 2). Figure 1 shows the HRM analysis of GADD45γ methylation.

No statistically significant difference was observed between DLBCL patients and controls in terms of GADD45γ methylation status (p=0.381). While the mean age was 48.56±22.69 years in the group that had methylated GADD45γ, it was 46.50±25.06 in the unmethylated group (p=0.716). Age status also did not significantly affect the methylation frequency of the GADD45γ gene (p=0.407). However, the methylation frequency in patients with advanced stage (stage 3 and 4) disease was 17 times higher than in early stages (stage 1 and 2), which was statistically significant (p=0.041). In addition, there was a difference in the methylation status of GADD45γ between nodal and extranodal involvement (p=0.040). The frequency of GADD45γ methylation in the group with high clinical risk (IPI score 3-4) was 2.6 times higher than that in the low clinical risk group (IPI score 0-2); however, this was not statistically significant (p=0.298) (Table 2).

### GADD45γ Protein Expression

**Figure 2:**
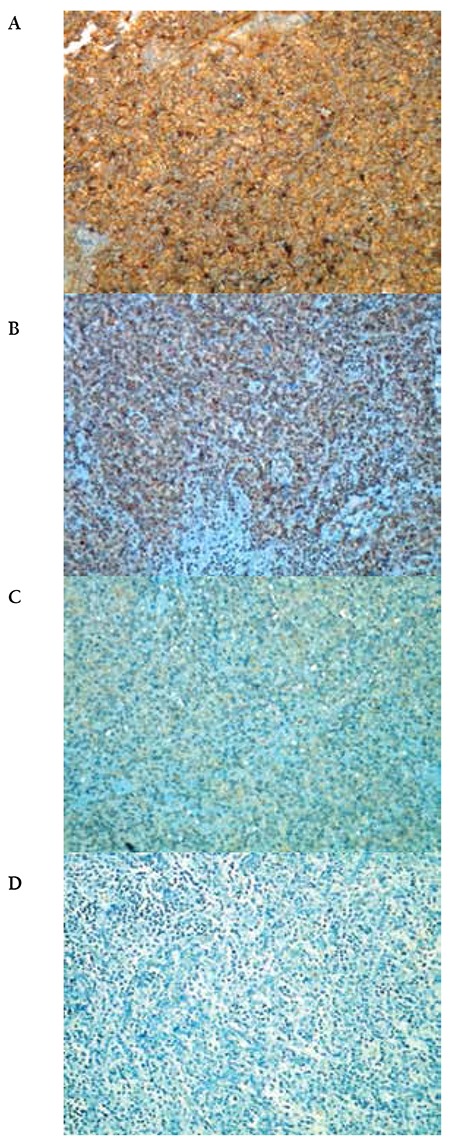
Representative immunohistochemical detection of GADD45γ in diffuse large B-cell lymphoma (A-D). Diffuse large B-cell lymphoma comprising large neoplastic lymphoid cells with strong GADD45γ staining intensity (Score: +++) (A), with moderate GADD45γ staining intensity (Score: ++) (B), with weak GADD45γ staining intensity (Score: +) (C), and with no GADD45γ staining (Score: 0) (D) (original magnification 200x).

GADD45γ protein expression was observed to be (0) in 1, (+) in 18, (++) in 11, and (+++) in 6 of the DLBCL cases. In controls, the numbers were 8, 30, and 2 for (0), (+), and (++), respectively. None of the controls were (+++) for GADD45γ protein expression. Since the numbers of samples in the subgroups were small, samples were combined for ease of statistical analysis. While (0) and (+) were regarded as low protein expression, (++) and (+++) were accepted as high protein expression (Figure 2). After this grouping, we found a statistically significant difference between DLBCL patients and controls (p<0.001) (Table 2).

High-level expression of GADD45γ was present in 37.5% of early and 50% of advanced stage DLBCL patients. There was no significant relation between the protein expression level and the stage of DLBCL (p=0.695). Similarly, we did not find a significant association between the protein expression level and other clinicopathologic parameters (Table 2).

### Association of GADD45γ Methylation Status with GADD45γ Protein Expression

Among 18 DLBCL patients with methylated GADD45γ, we observed the high expression and the low expression of GADD45γ in 8 (44.4%) and 10 (55.6%) patients, respectively. The high expression of GADD45γ was determined in 9 (50.0%) patients whose tumors had no methylated GADD45γ. Although we observed an association between GADD45γ methylation status and protein expression level in 48.7% of all patients included in this study, no significant correlation between the protein expression level and the status of methylation was observed (p=0.695) (Table 3).

## DISCUSSION

As major stress sensors of cells, GADD45 proteins might be key players of cancer development and progression. Although several studies have focused on the relationship between GADD45γ gene expression and methylation in hematologic malignancies and solid tumors, there are limited data in the literature about the involvement of GADD45γ methylation and protein expression in DLBCL development [13,26,27]. To our knowledge, this is the first study investigating the association between the methylation and the level of protein expression of GADD45γ in DLBCL patients and RLT controls. We detected GADD45γ methylation in 50.0% of DLBCL patients. MS-HRM used in this study was performed as previously described [26]. Zhang et al. found that the HRM protocol had high sensitivity, which allows the detection of low (1%) amounts of DNA methylation for GADD45γ [17]. It is well known that DNA derived from FFPE tissues is often degraded and the degradation of DNA is highly dependent on the sample age. In the present study, we used DNA samples extracted from FFPE tissues ranging in age from 2 to 5 years for MS-HRM. In a recent study, Kristensen et al. showed that DNA derived from up to 30-year-old FFPE tissue can be successfully used for DNA methylation analysis by MS-HRM [28]. Therefore, we suggest that MS-HRM analysis could be used to detect the methylation status of GADD45γ in FFPE tissue samples.

In non-small cell lung cancer, Na et al. reported that GADD45γ methylation was detected in 31.6% of cases. They also proposed that the silencing of GADD45γ by DNA methylation might be contributing to the development of lung cancer [29]. Bahar et al. detected GADD45γ methylation in 58% of human pituitary adenoma cases [27]. In 82% of patients whose tumors had no mRNA expression of GADD45γ, they detected promoter methylation by both methylation-specific PCR and sodium bisulfite sequencing. Ying et al. reported the epigenetic inactivation of GADD45γ in primary samples from various cancer types and tumor cell lines [13]. In their study, they found that GADD45γ methylation was more frequent in leukemia and lymphomas (16%-88%) than solid tumors (11%-16%). In their series, 38% of primary DLBCL tissues had GADD45γ promoter methylation, which is concordant with our results. Our results and theirs may be showing the specificity of GADD45γ methylation according to the epithelial or mesenchymal origin of tumors. Another interesting finding of our study was the increasing frequency of GADD45γ methylation with tumor progression. We found a significantly higher GADD45γ methylation frequency in advanced stages than early stages. This may show that the loss of function in the GADD45γ tumor suppressor gene by DNA methylation plays a key role in the progression of DLBCL.

It is well known that NHLs arise in different anatomical sites and they are considered as nodal and extranodal lymphomas according to the site [30,31]. The differences in clinical and biological characterizations between nodal and extranodal involvement are still not clear, as reflected in the heterogeneous nature of DLBCL in some sense, although there are a number of studies focused on the differences between lymphomas at different anatomical sites [32,33,34]. A recent study reported that primary extranodal involvement, especially at gastrointestinal, pulmonary, and liver/pancreatic sites, was associated with a worse outcome when compared to nodal involvement [35]. In our series, the majority of the patients had nodal involvement, while the remaining patients had both nodal and extranodal involvement. It was interesting that nodal involvement was observed in almost 80% of the patients with no methylated GADD45γ, with significant statistical difference, although there was no relation between the tissue involvement and IPI score. This finding may suggest that GADD45γ methylation status might be an important factor for the primary site of the lymphoma. Further studies are needed to identify the genetic and/or epigenetic differences between nodal and extranodal involvement in DLBCL.

There is no consensus in the literature about the relationship between GADD45γ methylation status and protein expression levels. Ying et al. found no GADD45γ expression in the cell lines with GADD45γ methylation in their above mentioned study [13]. Bahar et al. found a significant correlation between GADD45γ methylation and low protein expression, although there was expression of GADD45γ transcript in 9% of the patients with GADD45γ methylation [27]. Furthermore, 18% of patients without GADD45γ methylation did not have GADD45γ expression, either. In the present study, we could not find an association between GADD45γ methylation and protein expression in 51.3% of our cases. This finding may be explained by the following potential mechanisms: first, the method we used for the detection of methylation is not a quantitative method and those cases with GADD45γ expression might have low methylation levels that are not adequate for gene silencing. A number of studies have reported no significant association between protein expression and methylation status in different genes such as MGMT, DLC1, GATA4, NDK2, and RARRES1 [29,36]. It has also been reported that a gain of DNA methylation is not always associated with gene silencing. Kulis et al. characterized the DNA methylomes in patients with chronic lymphocytic leukemia and reported that there was a significant correlation between gene expression and DNA methylation levels in 4% of all CpGs [37]. In a study that identified DNA methylation differences in different human ethnic groups, it was shown that a gain of DNA methylation was associated with gene repression and activation in 63.0% and 37.0% of cases, respectively [38]. Second, our target in GADD45γ was relatively small because large amplicon sizes are generally unsuitable for HRM analysis. The GADD45γ gene has a unique CpG island that contains not only the promoter region but also exons [13,27]. Searching in the whole GADD45γ gene should be more accurate to detect the real methylation status. Third, since GADD45γ mutation was very rarely detected in primary tumors [13], the inhibition of expression might be due to other epigenetic mechanisms than DNA methylation, such as small noncoding RNAs and histone modifications. Finally, the polyclonal antibody that we used for IHC due to unavailability of commercial monoclonal antibody against GADD45γ protein might have cross-reacted with other epitopes in colocalized protein targets [39].

In summary, we found that the frequency of GADD45γ methylation in DLBCL was higher than that reported in solid tumors. We also observed that the frequency of GADD45γ methylation in advanced stages was significantly higher than that in early stages. In comparison to nodal DLBCL, GADD45γ was commonly methylated in extranodal DLBCL. These findings indicated that the silencing of GADD45γ by DNA methylation may play a role in the progression and the tissue involvement of DLBCL. Further studies are needed to evaluate the role of other members of the GADD45 family and their partners in DLBCL.

## Figures and Tables

**Table 1:**
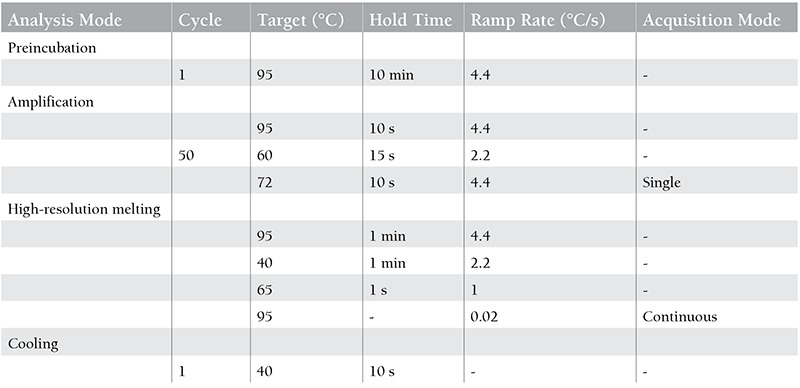
High-resolution melting protocol for GADD45g methylation.

**Table 2:**
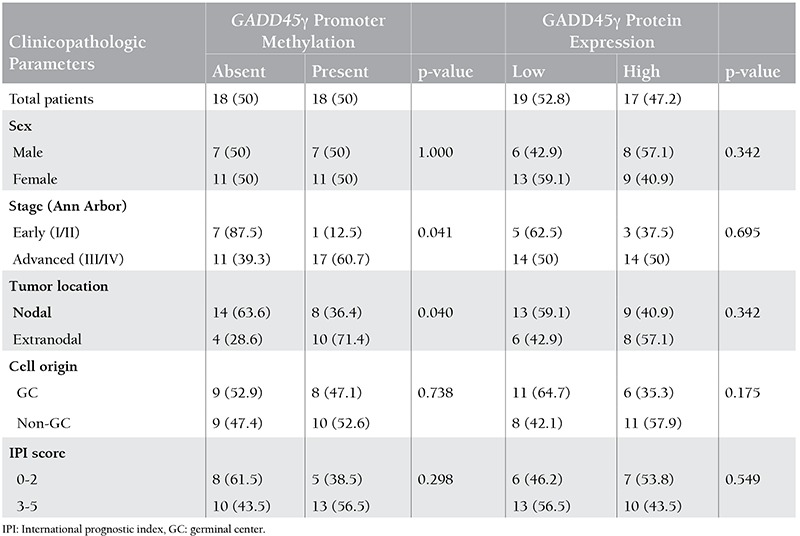
Associations of GADD45γ methylation and protein expression with clinicopathologic parameters in diffuse large B-cell lymphoma patients.

**Table 3:**
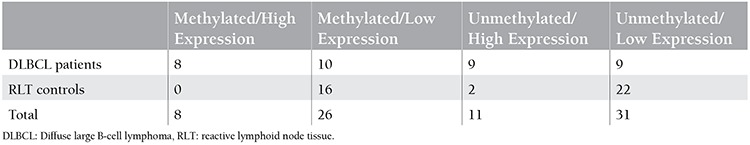
Association between GADD45γ methylation and its protein expression.
